# Interim Estimates of 2018–19 Seasonal Influenza Vaccine Effectiveness — United States, February 2019

**DOI:** 10.15585/mmwr.mm6806a2

**Published:** 2019-02-15

**Authors:** Joshua D. Doyle, Jessie R. Chung, Sara S. Kim, Manjusha Gaglani, Chandni Raiyani, Richard K. Zimmerman, Mary Patricia Nowalk, Michael L. Jackson, Lisa A. Jackson, Arnold S. Monto, Emily T. Martin, Edward A. Belongia, Huong Q. McLean, Angie Foust, Wendy Sessions, LaShondra Berman, Rebecca J. Garten, John R. Barnes, David E. Wentworth, Alicia M. Fry, Manish M. Patel, Brendan Flannery

**Affiliations:** ^1^Epidemic Intelligence Service, CDC; ^2^Influenza Division, National Center for Immunization and Respiratory Diseases, CDC; ^3^Baylor Scott & White Health, Texas A&M University Health Science Center College of Medicine, Temple, Texas; ^4^University of Pittsburgh Schools of the Health Sciences and University of Pittsburgh Medical Center, Pittsburgh, Pennsylvania; ^5^Kaiser Permanente Washington Health Research Institute, Seattle, Washington; ^6^University of Michigan School of Public Health, Ann Arbor, Michigan; ^7^Marshfield Clinic Research Institute, Marshfield, Wisconsin.

In the United States, annual vaccination against seasonal influenza is recommended for all persons aged ≥6 months (https://www.cdc.gov/flu/protect/whoshouldvax.htm). Effectiveness of seasonal influenza vaccine varies by season. During each influenza season since 2004–05, CDC has estimated the effectiveness of seasonal influenza vaccine to prevent laboratory-confirmed influenza associated with medically attended acute respiratory illness (ARI). This interim report uses data from 3,254 children and adults enrolled in the U.S. Influenza Vaccine Effectiveness Network (U.S. Flu VE Network) during November 23, 2018–February 2, 2019. During this period, overall adjusted vaccine effectiveness against all influenza virus infection associated with medically attended ARI was 47% (95% confidence interval [CI] = 34%–57%). For children aged 6 months–17 years, overall vaccine effectiveness was 61% (44%–73%). Seventy-four percent of influenza A infections for which subtype information was available were caused by A(H1N1)pdm09 viruses. Vaccine effectiveness was estimated to be 46% (30%–58%) against illness caused by influenza A(H1N1)pdm09 viruses. CDC recommends that health care providers continue to administer influenza vaccine because influenza activity is ongoing and the vaccine can still prevent illness, hospitalization, and death associated with currently circulating influenza viruses, or other influenza viruses that might circulate later in the season. During the 2017–18 influenza season, in which influenza A(H3N2) predominated, vaccination was estimated to prevent 7.1 million illnesses, 3.7 million medical visits, 109,000 hospitalizations, and 8,000 deaths (*1*). Vaccination can also reduce the severity of influenza-associated illness (*2*). Persons aged ≥6 months who have not yet been vaccinated this season should be vaccinated.

Methods used by the U.S. Flu VE Network have been published previously (*3*). At five study sites (Michigan, Pennsylvania, Texas, Washington, and Wisconsin), patients aged ≥6 months seeking outpatient medical care for an ARI with cough within 7 days of illness onset were enrolled. Study enrollment began after local surveillance identified increasing weekly influenza activity or one or more laboratory-confirmed cases of influenza per week for 2 consecutive weeks. Patients were eligible for enrollment if they met the following criteria: 1) were aged ≥6 months on September 1, 2018, and thus eligible for vaccination; 2) reported an ARI with cough with onset ≤7 days; and 3) had not been treated with influenza antiviral medication (e.g., oseltamivir) during this illness. After obtaining informed consent from patients or their guardians, participants or their proxies were interviewed to collect demographic data, information on general and current health status and symptoms, and 2018–19 influenza vaccination status. Nasal and oropharyngeal swabs (or nasal swabs alone for children aged <2 years) were collected to obtain respiratory specimens. Nasal and oropharyngeal swabs were placed together in a single tube of viral transport medium and tested at U.S. Flu VE Network laboratories using CDC’s real-time reverse transcription–polymerase chain reaction (real-time RT-PCR) protocol for detection and identification of influenza viruses. Participants (including children aged <9 years, who require 2 vaccine doses during their first vaccination season) were considered vaccinated if they received ≥1 dose of any seasonal influenza vaccine ≥14 days before illness onset, according to medical records and registries (at the Wisconsin site); medical records and self-report (at the Pennsylvania, Texas, and Washington sites); or self-report only (at the Michigan site). Vaccine effectiveness against all influenza virus types combined and against viruses by type/subtype was estimated as 100% x (1 - odds ratio).* Estimates were adjusted for study site, age group, sex, race/ethnicity, self-rated general health, number of days from illness onset to enrollment, and month of illness (4-week intervals) using logistic regression. Interim vaccine effectiveness estimates for the 2018–19 season were based on patients enrolled through February 2, 2019.

Among the 3,254 children and adults with ARI enrolled at the five study sites from November 23, 2018, through February 2, 2019, a total of 465 (14%) tested positive for influenza virus by real time RT-PCR, including 456 (98%) for influenza A viruses and nine (2%) for influenza B viruses (Table 1). Among 394 subtyped influenza A viruses, 293 (74%) were A(H1N1)pdm09 viruses, and 101 (26%) were A(H3N2) viruses. Of the eight influenza B viruses with lineage information available, four belonged to the B/Victoria lineage and four belonged to the B/Yamagata lineage. The proportion of patients with influenza differed by study site, age group, self-rated health status, and interval from illness onset to enrollment. The percentage of all ARI patients who were vaccinated ranged from 46% to 61% among study sites and differed by study site, sex, age group, race/ethnicity, and interval from illness onset to enrollment.

**TABLE 1:** Influenza test results and seasonal vaccination status among patients with medically attended acute respiratory illness (N = 3,254), by selected characteristics — U.S. Influenza Vaccine Effectiveness Network, November 23, 2018—February 2, 2019*

Characteristic	Test result status			Vaccination status^†^		
	Influenza-positive no. (%)	Influenza-negative no. (%)	P-value^§^	No. of patients	Vaccinated No. (%)	P-value^§^
**Overall**	465 (14)	2,789 (86)	—	3,254	1,789 (55)	—
**Study site**						
Michigan	76 (15)	438 (85)	0.006	514	314 (61)	<0.001
Pennsylvania	101 (17)	511 (83)		612	335 (55)	
Texas	72 (10)	637 (90)		709	327 (46)	
Washington	171 (16)	915 (84)		1,086	647 (60)	
Wisconsin	45 (14)	288 (86)		333	166 (50)	
**Sex**						
Male	208 (16)	1,128 (84)	0.08	1,336	704 (53)	0.03
Female	257 (13)	1,660 (87)		1,917	1,084 (57)	
**Age group**						
6 mos–8 yrs	118 (15)	689 (85)	0.03	807	453 (56)	<0.001
9–17 yrs	55 (19)	237 (81)		292	120 (41)	
18–49 yrs	166 (15)	932 (85)		1,098	461 (42)	
50–64 yrs	75 (13)	520 (87)		595	369 (62)	
≥65 yrs	51 (11)	411 (89)		462	386 (84)	
**Race/Ethnicity^¶^**						
White	296 (14)	1,895 (86)	0.33	2,191	1,275 (58)	<0.001
Black	63 (17)	318 (83)		381	165 (43)	
Other race	53 (15)	290 (85)		343	199 (58)	
Hispanic	51 (16)	277 (84)		328	143 (44)	
**Self-rated health status**						
Fair/Poor	21 (9)	223 (91)	0.003	244	145 (59)	0.12
Good	105 (13)	723 (87)		828	475 (57)	
Very good	177 (14)	1,050 (86)		1,227	651 (53)	
Excellent	162 (17)	791 (83)		953	517 (54)	
**Illness onset to enrollment (days)**						
<3	191 (19)	795 (81)	<0.001	986	509 (52)	0.01
3–4	176 (15)	1,037 (85)		1,213	666 (55)	
5–7	98 (9)	957 (91)		1,055	614 (58)	
**Influenza test result**						
Negative	—	2,789	—	2,789	1,591 (57)	—
Influenza B positive	9 (2)	—		9	3 (33)	
B/Yamagata	4 (50)**	—		4	2 (50)	
B/Victoria	4 (50)**	—		4	1 (25)	
B lineage pending	1 (—)	—		1	0 (0)	
Influenza A positive	456 (98)	—		456	195 (43)	
A (H1N1)pdm09	293 (74)^††^	—		293	125 (43)	
A (H3N2)	101 (26)^††^	—		101	42 (42)	
A subtype pending	62 (—)	—		62	28 (45)	

* Sex was unknown for one patient, race/ethnicity for 11 patients, and self-rated health status for two patients.

^†^ Defined as having received ≥1 dose of influenza vaccine ≥14 days before illness onset. A total of 78 participants who received the vaccine ≤13 days before illness onset were excluded from the study sample.

^§^ The chi-square statistic was used to assess differences between the numbers of persons with influenza-negative and influenza-positive test results, in the distribution of enrolled patient and illness characteristics, and in differences between groups in the percentage vaccinated.

^¶^ Patients were categorized into one of four mutually exclusive racial/ethnic populations: white, black, other race, and Hispanic. Persons identifying as Hispanic might have been of any race. Persons identifying as white, black, or other race were non-Hispanic.

** Percentage for which lineage information was available (n = 8).

^††^ Percentage for which subtype information was available (n = 394).

Among participants, 43% of those with influenza had received the 2018–19 seasonal influenza vaccine, compared with 57% of influenza-negative participants (Table 2). The adjusted vaccine effectiveness against medically attended ARI caused by all influenza virus types combined was 47% (95% CI = 34%–57%). Vaccine effectiveness for all ages was 46% (30%–58%) against medically attended ARI caused by A(H1N1)pdm09 virus infection and 44% (13%–64%) against influenza A(H3N2) virus infection. Among children aged 6 months–17 years, vaccine effectiveness against all influenza virus types was 61% (44%–73%), and effectiveness against influenza A(H1N1)pdm09 was 62% (40%–75%). Among adults ≥50 years, vaccine effectiveness against all influenza types and influenza A(H1N1)pdm09 was 24%(-15% to 51%) and 8% (-59% to 46%), respectively; neither were significant.

**TABLE 2:** Number and percentage outpatients with acute respiratory illness and cough (N = 3,254) receiving 2018–19 seasonal influenza vaccine, by influenza test result status, age group, and vaccine effectiveness* against all influenza A and B and against virus type A(H1N1)pdm09 — U.S. Influenza Vaccine Effectiveness Network, November 23, 2018–February 2, 2019

Influenza type/Age group	Influenza-positive		Influenza-negative		Vaccine effectiveness*	
	Total	Vaccinated no. (%)	Total	Vaccinated no. (%)	Unadjusted % (95% CI)	Adjusted % (95% CI)^†^
**Influenza A and B**						
Overall	465	198 (43)	2,789	1,591 (57)	44 (32 to 54)	47 (34 to 57)^§^
Age group						
6 mos–17 yrs	173	58 (34)	926	515 (56)	60 (43 to 71)	61 (44 to 73)^§^
18–49 yrs	166	58 (35)	932	403 (43)	30 (1 to 50)	37 (9 to 56)^§^
≥50 yrs	126	82 (65)	931	673 (72)	29 (-6 to 52)	24 (-15 to 51)
**Influenza A(H3N2)**						
Overall	101	42 (42)	2,789	1,591 (57)	46 (20 to 64)	44 (13 to 64)^§^
**Influenza A(H1N1)pdm09**						
Overall	293	125 (43)	2,789	1,591 (57)	44 (29 to 56)	46 (30 to 58)^§^
Age group						
6 mos–17 yrs	106	37 (35)	926	515 (56)	57 (35 to 72)	62 (40 to 75)^§^
18–49 yrs	113	38 (34)	932	403 (43)	33 (0 to 56)	45 (14 to 64)^§^
≥50 yrs	74	50 (68)	931	673 (72)	20 (-33 to 52)	8 (-59 to 46)

* Vaccine effectiveness was estimated as 100% x (1 - odds ratio [ratio of odds of being vaccinated among outpatients with influenza-positive test results to the odds of being vaccinated among outpatients with influenza-negative test results]); odds ratios were estimated using logistic regression.

^†^ Adjusted for study site, age group, sex, race/ethnicity, self-rated general health, number of days from illness onset to enrollment, and month of illness (4-week intervals) using logistic regression.

^§^ Statistically significant at p<0.05.

## Discussion

Influenza activity remains elevated in the United States (*4*). Overall, influenza A(H1N1)pdm09 viruses have predominated in most of the country, although circulation of influenza A(H3N2) and low levels of influenza B viruses have also been observed. Effectiveness of influenza vaccines in reducing the risk for medically attended influenza illness has ranged from approximately 40%–60% across all ages during seasons when most circulating influenza viruses are antigenically like the recommended influenza vaccine components. The overall interim estimate of 47% vaccine effectiveness against influenza A(H1N1)pdm09 in all age groups is similar to that observed during the most recent A(H1N1)pdm09 predominant season (45%) in 2015–16 (*3*), but lower than a meta-analysis of vaccine effectiveness against A(H1N1)pdm09 since the 2010–11 season in the United States (*5*). This interim estimate also is lower than the recently reported interim estimates of 72% effectiveness against A(H1N1)pdm09 in Canada during the 2018–19 season (*6*) and 78% against A(H1N1)pdm09 in Australia during the 2018 Southern Hemisphere influenza season (*7*). The reasons for these differences might include limited sample size caused by low attack rates in some age groups, geographic differences in circulating viruses, and genetic variation within virus subtypes (*4*). Of note, vaccine effectiveness against A(H1N1)pdm09 among children and adolescents aged 6 months–17 years (62%) was similar to that observed during the 2015–16 season in this age group (49%–63%) (*3*). Among adults aged ≥50 years, interim estimates of effectiveness were not significant. Vaccine effectiveness against A(H3N2) virus infection was 44% (95% CI = 13%–64%) but a limited number of A(H3N2) viruses were detected. Several more weeks of influenza are likely, and CDC continues to recommend influenza vaccination while influenza viruses are circulating in the community. Vaccination can protect against infection with influenza viruses that are currently circulating, as well as those that may circulate later in the season.

Vaccination remains the best method for preventing influenza and its potentially serious complications, including those that can result in hospitalization and death. In particular, vaccination has been found to reduce the risk for influenza-associated deaths in children (*8*). During past seasons, including the 2017–18 season, approximately 80% of reported pediatric influenza-associated deaths have occurred in children who were not vaccinated. Vaccination also has been found to reduce the risk for influenza-associated hospitalization in pregnant women (*9*) and can reduce the risk for cardiac events among persons with heart disease (*10*). CDC recommends antiviral treatment for any patient with suspected or confirmed influenza who is hospitalized, has severe or progressive illness, or is at high risk for complications from influenza, regardless of vaccination status or results of point-of-care influenza diagnostic tests.^†^ Antiviral treatment also can be considered for any previously healthy symptomatic outpatient not at high risk for complications, with confirmed or suspected influenza, if treatment can be started within 48 hours of illness onset.

The findings in this report are subject to at least four limitations. First, sample sizes are smaller than in recent interim reports, resulting in wide confidence intervals, particularly in adults aged ≥50 years. The small sample size also limits the number of age groups included in this analysis. This limitation is common among interim vaccine effectiveness reports during mild or late influenza seasons. End-of-season vaccine effectiveness estimates could change as additional patient data become available or if a change in circulating viruses occurs later in the season. Second, vaccination status included self-report at four of five sites; end-of-season vaccine effectiveness estimates based on updated documentation of vaccination status might differ from interim estimates. For this reason, the type of vaccine received by participants (e.g., egg-based, cell culture–based, or recombinant antigen) is not available at this time, although this information will be updated at the end of the season. Third, an observational study design has greater potential for confounding and bias than do randomized clinical trials. However, the test-negative design is widely used in vaccine effectiveness studies and has been used by the U.S. Flu VE Network to estimate vaccine effectiveness for previous influenza seasons. Finally, the vaccine effectiveness estimates in this report are limited to the prevention of outpatient medical visits rather than more severe illness outcomes, such as hospitalization or death; data from studies measuring vaccine effectiveness against more severe outcomes will be available at a later date.

Vaccination prevents a substantial number of influenza-related illnesses, hospitalizations, and deaths annually. However, better protection and improved vaccination coverage are needed to realize the full potential of influenza vaccines. Evaluation of influenza vaccine effectiveness is an essential component of ongoing efforts to improve influenza vaccines. Influenza activity remains elevated in the United States, highlighting the importance of vaccination. CDC will continue to monitor influenza disease throughout the season to better understand the impact of vaccination, identify factors associated with reduced protection, and support efforts to improve influenza vaccines.

**Summary**

**What is already known about this topic?**

Annual vaccination against seasonal influenza is recommended for all U.S. persons aged ≥6 months. Effectiveness of seasonal influenza vaccine varies by season.

**What is added by this report?**

On the basis of data from the U.S. Influenza Vaccine Effectiveness Network on 3,254 children and adults with acute respiratory illness during November 23, 2018–February 2, 2019, the overall estimated effectiveness of seasonal influenza vaccine for preventing medically attended, laboratory-confirmed influenza virus infection was 47%.

**What are the implications for public health practice?**

Vaccination remains the best way to protect against influenza and its potentially serious complications. CDC continues to recommend influenza vaccination while influenza viruses are circulating in the community.

## Footnotes

* 100% x (1 – odds ratio [ratio of odds of being vaccinated among outpatients with influenza-positive test results to the odds of being vaccinated among outpatients with influenza-negative test results]).

† A complete summary of guidance for antiviral use is available at https://www.cdc.gov/flu/professionals/antivirals/summary-clinicians.htm. Groups at high risk for influenza complications include the following: children aged <2 years; adults aged ≥65 years; persons with chronic pulmonary conditions (including asthma); persons with cardiovascular disease (except hypertension alone); persons with renal, hepatic, or hematologic (including sickle cell) disease; persons with metabolic disorders (including diabetes mellitus); persons with neurologic and neurodevelopmental conditions (including disorders of the brain, spinal cord, peripheral nerves and muscles, such as cerebral palsy, epilepsy [seizure disorders], stroke, intellectual disability [mental retardation], moderate to severe developmental delay, muscular dystrophy, or spinal cord injury); persons with immunosuppression, including that caused by medications or by human immunodeficiency virus infection; women who are pregnant or ≤2 weeks postpartum; persons aged <19 years who are receiving long-term aspirin or salicylate-containing medications; American Indian/Alaska Natives; persons with morbid obesity (i.e., body-mass index ≥40); and residents of nursing homes and other chronic-care facilities.

## References

[1] Rolfes MA, Flannery B, Chung J, Effects of influenza vaccination in the United States during the 2017–2018 influenza season. Clin Infect Dis 2019. Epub February 2, 2019. 10.1093/cid/ciz075 PMC7188082 30715278

[2] Arriola C, Garg S, Anderson EJ, Influenza vaccination modifies disease severity among community-dwelling adults hospitalized with influenza. Clin Infect Dis 2017;65:1289–97. 10.1093/cid/cix468 28525597 PMC5718038

[3] Jackson ML, Chung JR, Jackson LA, Influenza vaccine effectiveness in the United States during the 2015–2016 season. N Engl J Med 2017;377:534–43. 10.1056/NEJMoa1700153 28792867 PMC5727917

[4] Blanton L, Dugan VG, Elal AIA, Update: influenza activity—United States, September 30, 2018–February 2, 2019. MMWR Morb Mortal Wkly Rep 2019;68:125–34. 30763296 10.15585/mmwr.mm6806a1 PMC6375659

[5] Belongia EA, Simpson MD, King JP, Variable influenza vaccine effectiveness by subtype: a systematic review and meta-analysis of test-negative design studies. Lancet Infect Dis 2016;16:942–51. 10.1016/S1473-3099(16)00129-8 27061888

[6] Skowronski DM, Leir S, Sabaiduc S, Interim estimates of 2018/19 vaccine effectiveness against influenza A(H1N1)pdm09, Canada, January 2019. Euro Surveill 2019;24:1900055. 10.2807/1560-7917.ES.2019.24.4.1900055 30696523 PMC6351998

[7] Australian Government Department of Health. Information brief: 2018 influenza season in Australia. Canberra, Australia: Australian Government Department of Health; 2016. http://www.health.gov.au/internet/main/publishing.nsf/Content/cda-surveil-ozflu-flucurr.htm/$File/2018-Season-Summary.pdf

[8] Flannery B, Reynolds SB, Blanton L, Influenza vaccine effectiveness against pediatric deaths: 2010–2014. Pediatrics 2017;139:e20164244. 10.1542/peds.2016-4244 28557757 PMC5728382

[9] Thompson MG, Kwong JC, Regan AK, Influenza vaccine effectiveness in preventing influenza-associated hospitalizations during pregnancy: a multi-country retrospective test negative design study, 2010–2016. Clin Infect Dis 2018. Epub October 11, 2018. 10.1093/cid/ciy737 30307490

[10] Udell JA, Zawi R, Bhatt DL, Association between influenza vaccination and cardiovascular outcomes in high-risk patients: a meta-analysis. JAMA 2013;310:1711–20. 10.1001/jama.2013.279206 24150467

